# Alternative RNA Splicing in Fatty Liver Disease

**DOI:** 10.3389/fendo.2021.613213

**Published:** 2021-02-26

**Authors:** Panyisha Wu, Moya Zhang, Nicholas J. G. Webster

**Affiliations:** ^1^ VA San Diego Healthcare System, San Diego, CA, United States; ^2^ Department of Medicine, Division of Endocrinology and Metabolism, University of California San Diego, La Jolla, CA, United States; ^3^ Moores Cancer Center, University of California, San Diego, La Jolla, CA, United States; ^4^ University of California Los Angeles, Los Angeles, CA, United States

**Keywords:** RNA splicing, splicing factor, NAFLD, NASH, cirrhosis

## Abstract

Alternative RNA splicing is a process by which introns are removed and exons are assembled to construct different RNA transcript isoforms from a single pre-mRNA. Previous studies have demonstrated an association between dysregulation of RNA splicing and a number of clinical syndromes, but the generality to common disease has not been established. Non-alcoholic fatty liver disease (NAFLD) is the most common liver disease affecting one-third of adults worldwide, increasing the risk of cirrhosis and hepatocellular carcinoma (HCC). In this review we focus on the change in alternative RNA splicing in fatty liver disease and the role for splicing regulation in disease progression.

## Introduction

Eukaryotic protein-coding genes are usually split into exons and intervening introns that are removed by the process of RNA splicing (1). Alternative splicing is the process that selectively removes introns or exons, or parts thereof, to generate multiple messenger RNAs (mRNAs) from a single precursor mRNA (pre-mRNA) (2). First reported in 1980, RNA alternative splicing events have been found in the majority of eukaryotic genes. Indeed, it is estimated that more than 95% of the genes in the human cell undergo alternative splicing and produce isoforms of different or even opposing biological roles (3). Therefore, alternative RNA splicing plays a critical role in defining the transcriptome and fine-tuning the proteome of the cell.

Recent studies have shown that changes in RNA splicing and RNA binding protein expression occur during the maturation of the liver (4, 5), are associated with aging and sexual dimorphism in the liver (6), and have also been documented in hepatocellular carcinoma (7–16), but little is known about changes in RNA splicing in early liver disease (17). Non-alcoholic fatty liver disease (NAFLD) is the leading cause of liver disease in western countries, affecting almost 25% of the adult population in the world (18). It is defined as the fat accumulation in the liver after the exclusion of secondary causes (19). NAFLD can progress from simple steatosis to non-alcoholic steatohepatitis (NASH), cirrhosis and even hepatocellular carcinoma (HCC) (20). Not surprisingly, NAFLD is strongly associated with the metabolic syndrome (21, 22) and the prevalence of NAFLD is rising due to the global obesity epidemic (23). Yet the risk of NAFLD is affected by both environmental and genetic factors (24). Many studies have assessed gene expression changes in NAFLD patients and identified transcripts that are associated with specific metabolic comorbidities in patients with NAFLD and NASH (25–28). Changes in splicing factor expression have also been observed which would suggest alterations in RNA splicing (29, 30). Furthermore, some studies have demonstrated alternative splicing of obesity-related genes in the liver and other metabolic tissues, suggesting that splicing variants may play a role in NAFLD development (31–35). Thus, the evidence to date points to a potential association between liver disease and RNA splicing that deserves further investigation. In this article, we review prior evidence and discuss more recent reports of alternative RNA splicing events and their significance in the process of fatty liver disease.

## Regulation of Alternative Splicing

The human genome, as well as many other eukaryotic genomes, contains stretches of intronic sequence between sparser and relatively smaller, exonic sequences. The process of removing the introns from the pre-mRNA is called RNA splicing and has been extensively reviewed (2, 36–38). Of interest here, these introns can be spliced out in the pre-mRNA at different locations and efficiencies to form different arrangements of exons in the final mRNA transcripts through the use of alternative splice sites (2). This process allows for greater RNA and protein diversity than would be predicted by the number of genes (3, 39). Briefly, a complex RNA-based molecular machine called the spliceosome recognizes the splice sites and performs the intron excision and exon joining (40). The spliceosome is formed from five main subunits of small nuclear ribonucleoprotein particles (snRNPs) (41, 42). In canonical splicing, the U1 snRNP recognizes a GU-containing hexamer sequence at the 5′ site of the intron, while the U2 subunit in combination with an auxiliary factor U2AF recognizes the branch-point sequences, a pyrimidine-rich sequence, and the AG-splice site at the 3′ site of the intron. Once these molecules are bound to the RNA, they recruit the U4/U5/U6 snRNPs to form the spliceosome which then rearranges to form an active splicing complex. This complex cleaves the intron at the 5′ and 3′ ends then ligates the exons together by a trans-esterification mechanism (7, 43). While a U1 and U2 interaction across the intron (intron definition) has been demonstrated *in vitro*, it is only effective when the introns are relatively short (less than 250 nucleotides). For the majority of long introns *in vivo*, this U1–U2 splice site recognition process is thought to occur across exons, in a process called exon definition (43).

Recognition of 5′ splice sites is mediated by base pairing of the U1 snRNA, a component of the U1 snRNP, with the sequences surrounding the splice site, whereas the 3′ splice site is recognized in a less sequence specific manner by U2AF binding to the polypyrimidine tract at the 3′ splice site allowing the U2snRNA to base-pair with the branch-point sequence. As such, any divergence of the splice site sequence from the consensus will weaken recognition by the snRNPs and use of the splice site by the spliceosome. This allows for selection of alternate exons, or the use of alternative splice sites in exons. Recognition of these weak splice sites is modulated through the binding of RNA-binding proteins (RBPs) to adjacent short regulatory sequences. These sequences can either repress or enhance splicing at the adjacent site. Many RBPs are involved in alternative splicing, including the serine/arginine-rich (SR) proteins and the heterogeneous nuclear ribonucleoprotein (hnRNPs) families, that together target the spliceosome to the appropriate splice site (7). As a general, but not hard-and-fast rule, SR proteins are typically involved with exonic splicing enhancers (ESEs) and help stabilize the snRNPs at splice sites (44, 45), but hnRNPs interact with exonic splicing silencers (ESSs) and prevent the binding of SR proteins or snRNPs. The hnRNPs thus mainly play a role in exon skipping, in which the exon and its neighboring introns are spliced out of the pre-mRNA (7, 44).

Extracellular factors have also been shown to contribute to the regulation of alternative splicing (46). Extracellular stimuli can affect the phosphorylation of splicing factors, either promoting or inhibiting binding to the RNA (47). Kinases and phosphatases that perform post-translational modifications of splicing factors have also been identified (7, 46). These different mechanisms expand the versatility of alternative splicing, allowing the body to produce a wide variety of proteins and molecules based off of a relatively limited genome.

## Global Changes in the Splicing Machinery in NAFLD

To understand the factors involved in the development of NAFLD, transcriptome profiling of human livers has been performed by microarray and RNAseq, and changes in the expression of many transcription factors have been documented, such as *FOXO1, SREBP1, IRF1, IRF3, C/EBPβ, SMAD3, SMAD7, PPARα* and *PPARβ* (48–51); however, most of the studies did not investigate changes in RNA splicing factors or spliceosome components. In recent years, in recognition of the increasingly important role for RNA splicing, a number of studies (10, 29, 48, 52) have investigated whether the components of the alternative splicing machinery may be altered in NAFLD and NASH that might underlie changes in splicing variants (**Table 1**).

**Table 1:** Studies reporting alterations of RNA splicing components in NAFLD or NASH.

Study	Objective	Method	Ref
**Wang et al.**	Splicing factor expression in NAFLD and AFLD	Western blot for 10 splicing factors in mouse models of NAFLD, AFLD, inflammation, fibrosis. Confirmed in 152 paired HCC normal human samples.	(10)
**Starmann et al.**	Comparison of liver transcriptomes in steatosis and steatohepatitis in humans	Microarrays on 10 healthy controls, 14 steatosis and 8 steatohepatitis.	(25)
**Arendt et al.**	Comparison of transcriptomes in steatosis and NASH	Microarrays on 24 healthy liver, 20 individuals with steatosis, and 19 with NASH	(27)
**Tuefel et al.**	Comparison of liver transcriptomes in mouse models of NAFLD with human NAFLD or NASH	Microarrays on C57BL/6 mice, and 25 obese, 27 NAFLD, 25 NASH, and 39 normal human subjects	(28)
**Del Rio-Moreno et al.**	Profile splicing factor machinery in women with steatosis	Qunatitative PCR in 32 obese women and 9 obese women with hepatic steatosis	(29)
**Pihlajamaki et al.**	Comparison of liver transcriptomes in obese and lean humans and mice	Microarrays on 5 lean non-diabetics and 8 obese subjects undergoing bariatric surgery	(30)
**Gerhard et al.**	Comparison of liver transcriptomes in individuals with normal liver histology, lobular inflammation, or advanced fibrosis	RNAseq on 24 normal, 53 lobullar inflammation and 65 bridging fibrosis, incomplete cirrhosis, or cirrhosis	(48)
**Suppli et al.**	Comparison of liver transcriptomes in obesity, NAFLD and NASH in humans	RNAseq on 14 normal, 12 obese healthy, 15 NAFLD, and 16 NASH	(49)
**Hoang et al.**	Comparison of liver transcriptomes in individuals with varying degrees of NAFLD	RNAseq of 6 normal liver and 72 biopsy-confirmed NAFLD	(50)
**Zhu et al.**	Liver transcriptome and alcohol metabolizing genes in NAFLD	Microarrays on 40 mild NAFLD, 32 severe NAFLD, 15 alcoholic hepatitis, and 7 normal subjects	(52)
**Ye et al.**	NAFLD transcriptional networks in humans	Microarrays on 10 steatotic, 16 NASH, and 19 normal subjects	(53)
**Lake at al.**	Transcriptome of NAFLD	Microarrays on 10 steatotic, 9 NASH with fatty liver, 7 NASH w/o fatty liver, and 19 normal subjects	(54)
**Bangru et al.**	Transcriptome changes during liver regeneration	RNAseq on mice treated with DDC to cause liver injury	(55)
**Almanza et al.**	Comparison of liver transcriptome in mouse model of NAFLD	RNAseq on SAMP6 mice fed a high-fat diet to induce NAFLD	(56)
**Kristiansen et al.**	Comparison of liver transcriptome in mouse model of NASH	RNAseq on C57BL/6 mice fed a high-trans-fat, high-cholesterol diet to induce NASH	(57)
**Van Koppen et al.**	Comparison of liver transcriptome in mouse model of NASH	RNAseq on LDLR KO mice fed a high-fat diet to induce NASH	(58)

The association of splicing factors with obesity and NAFLD was initially investigated by microarray analysis (53, 59, 60), which allowed quantification of gene expression but not RNA splicing. NAFLD is strongly associated with obesity, and several obesity-linked genes have been shown to be regulated by alternative splicing (61–64); therefore, Pihlajamaki et al. identified differentially expressed genes in the liver and muscle biopsies from obese patients by high-density oligonucleotide arrays (30). Five lean control subjects and eight obese subjects were included for the liver cohort. Though none of them had abnormalities in glucose metabolism at the start of the study, six of the obese subjects were diagnosed with type 2 diabetes mellitus (T2D) during the study. When they analyzed the pathways affected by the alterations in expression, they found the top two pathways were mRNA processing and RNA splicing pathways. Forty-six of the 199 analyzed RNA splicing genes were downregulated in obese livers, and the expression of 13 genes decreased in both liver and muscle, including *SFRS10 (TRA2b), SFRS7 (9G8), SF3A1, SFRS2 (SC35), SFPQ, HNRPA1, HNRPK*. The decreased expression of *SFRS10, SFRS7, SF3A1, SFPQ* and *HNRPK* in obese liver was further confirmed in a mouse model of diet-induced obesity. This study demonstrated that RNA splicing factor expression inversely correlated with hepatic fat accumulation and hyperinsulinemia and that alterations in RNA splicing factor expression may contribute to obesity-related phenotypes.

Starmann et al. (25) profiled healthy controls (n = 10) and patients with simple steatosis (n = 14) or steatohepatitis (n = 8) by microarray. They found 4,963 genes altered in the steatohepatitis patients *versus* healthy liver or 2,542 genes altered *versus* the simple steatosis patients. Inspection of these differentially expressed genes for known splicing factors showed that 136 splicing factors were altered compared to healthy liver and 41 compared to simple steatosis. Among the genes altered were eight *HNRNPs (A2B1, H1/2/3, L, F, D & U), RBFOX2*, eight *RBMs (12B, 14, 22, 7, 10, 20, 4 & 6)*, four *SF3* genes (*B6, B5, A3, & B2*), *SLU7, SFPQ, SRSF11, MBNL3*, and *TRA2A*.

Zhu et al. (52) examined hepatic gene expression in 72 patients with mild NAFLD (fibrosis stage 0–1, n = 40) and severe NAFLD (fibrosis stages 3–4, n = 32), alcoholic hepatitis (AH, n = 15), or healthy liver (n = 7) by microarray. The mild and severe NAFLD and AH clustered together but were distinct from the healthy controls. Although not addressed in the paper, reanalysis of their dataset showed that the expression of 92 splicing factor genes was altered in subjects with mild NAFLD *versus* healthy controls. Among these altered genes were *ESRP1/2, MBNL1/2/3, SLU7*, nine *SF3* genes and ten *SRSF* proteins.

Ye et al. (53) used weighted gene co-expression network analysis on a NASH-NAFLD microarray dataset generated by Lake et al. (54) and found that modules involved in RNA processing with enrichment for genes involved in RNA binding, mRNA processing and the spliceosome in the NASH and NAFLD groups.

More recently in 2018, Gerhard et al. (48) performed RNAseq of liver samples from individuals with normal histology (n = 24), lobular inflammation (n = 53), or advanced fibrosis, defined by bridging fibrosis, incomplete cirrhosis, or cirrhosis (n = 65). They reported differential expression of 3,820 and 2,980 genes in the lobular inflammation and advanced fibrosis groups compared to normal histology. In addition to genes involved in inflammation, extracellular matrix, cytokine and PI-3K signaling, and focal adhesion, 35 splicing factors were altered in the lobular inflammation group, including *ESRP1, RBM4/20/24, SF3B5, HNRNPU, CELF3/4/5, ELAVL2/4, NOVA1/2*, and *RBFOX1/3*, and 20 were altered in the advanced fibrosis group including *CELF3/4/5, ELAVL2/4, RBM20/24, NOVA1/2*, and *RBFOX1/3*.

To specifically address whether the splicing machinery is altered in steatosis, Del Rio-Moreno et al. (29) profiled the expression of spliceosome components and splicing factors in liver samples obtained from 41 obese women with (n = 32) and without (n = 9) hepatic steatosis by qPCR. The patients with steatosis were further classified into mild, moderate, or severe steatosis by liver echography. It should be noted that all the patients in this study presented with steatosis at an early stage of NAFLD without any evidence of NASH or cirrhosis. The expression of 17 splicing machinery components and 28 splicing factors was determined. It was found that the expression of 16 of these 45 genes was clearly different between patients with and without hepatic steatosis, including eight spliceosome components (*RNU6ATAC, RNU6, SF3B1, RNU2, RNU4ATAC, RBM22, U2AF1, U2AF2)* and eight splicing factors (*PTBP1, SRRM1, SND1, KHDRSB1, SRSF2, SRSF10, ESRP2, TIA1).* In patients with steatosis the expression of *RNU6ATAC, RNU6, SF3B1, RNU2, RNU4ATAC*, *TIA1* was downregulated, but the expression of the other 10 genes was elevated significantly. When the patients with steatosis were grouped according to similar expression patterns of spliceosome components and splicing factors, patients in Cluster A (characterized by lower *SRSF4* and *TRA2B*) showed increased blood glucose and haptoglobin levels, whereas patients in Cluster B (higher *RBM45* and *TRA2A*) had higher plasma triglycerides, GGT, and lower alkaline phosphatase levels, and Cluster C (higher *SND1* and *RAVER1*) exhibited elevated insulin, ALT, and AST levels. Moreover, Cluster C presented a worse response to bariatric surgery, compared with Clusters A+B, exhibiting less normalization of plasma GGT, glucose, triglycerides, alkaline phosphatase, and HDL levels. The differences of these three molecular NAFLD phenotypes suggest that the dysregulation of specific splicing machinery components is associated with distinct clinical/metabolic alterations. To test whether a causal relationship could exist, the authors used RNAi knockdown in HepG2 cells. Knockdown of specific splicing factors (*PTBP1, SRSF4, RBM22, RBM45, SND1, RAVER1*) significantly lowered lipid accumulation in HepG2 hepatoma cells after lipid loading with oleic acid. Although expression of the selected splicing factors was not altered by oleic acid treatment, the expression of some splicing machinery components was modulated by other metabolic factors. For example, elevated glucose decreased *SND1*, and leptin decreased *RBM22*, but insulin-like growth factor 1 (*IGF-1*) increased *RAVER1*, and palmitic acid increased *PTBP1* and *RBM22*. Thus, this study provided evidence that not only could the overexpression of *SRSF4, RBM45, SND1*, and *RAVER1* that is seen in the three molecular clusters enhance the development of NAFLD, but also that the metabolic milieu could contribute to the altered RNA splicing.

In 2019, Suppli et al. (49) also published an RNAseq study on liver samples from healthy normal (n = 14), obese individuals (n = 12), and NAFLD (n = 15) and NASH (n = 16) patients. They found that genes involved in RNA metabolism were enriched in samples from NASH patients compared to NAFLD. Although many genes (8,244) were differentially expressed in NAFLD and NASH compared to healthy liver, the authors did not provide the lists of differentially expressed genes and did not investigate whether RNA splicing genes were altered. A reanalysis of these data, however, showed that many RNA splicing factor genes were altered in NAFLD (174) and NASH (204).

That same year, Hoang et al. (50) published a study of 72 patients with varying degrees of biopsy-confirmed NAFLD compared to six healthy controls by RNAseq. Patients’ samples were assessed by NAFLD activity score (NAS) or fibrosis stage. The authors used ordinal regression to identify genes that significantly changed with severity of disease either by NAS or fibrosis stage. At a false discovery rate of 1%, they identified 2,970 genes associated with NAS and 1,656 genes associated with fibrosis stage. Integration of these genes with protein-interaction networks demonstrated that genes involved in immune signaling, extracellular matrix organization, and cell cycle were enriched. They also identified genes enriched in RNA metabolism associated with both NAS and fibrosis stage. Inspection of the list of significant genes shows that 88 splicing factors are associated with NAS and 52 with fibrosis stage, suggesting a change in the splicing machinery with disease progression.

Similar to the human data, there is evidence that splicing factor expression is altered in different liver disease stages in six mouse models of non-alcoholic and alcoholic fatty liver disease (AFLD). In a recent study, Wang et al. (10) evaluated the expression of 10 splicing factors (PSF, NONO, SRSF1, SRSF3, SRSF6, SRSF7, hnRNPA2B1, hnRNPH, La, and SF1) in mouse livers by western blot. A significant decrease of SRSF3 and increases of NONO, SRSF6, hnRNPA2B1 and hnRNPH protein were detected in livers of HFD-induced NAFLD mice. The AFLD mouse model also showed increased expression of PSF, p47, SRSF7 and La. To model the effect of disease progression, male mice were injected with LPS and CCl_4_ to induce liver inflammation and fibrosis, respectively. As a result, the level of p47, SRSF3, SRSF6 and La was upregulated in the LPS-induced inflammatory livers, while SRSF6, SRSF7, and SF1 levels were elevated in fibrotic livers. Furthermore, they confirmed alteration in many of these splicing factors in RNA from 152 paired human HCC and normal samples.

Consistent with the known developmental changes in RNA splicing during liver maturation, liver injury caused by 0.1% 3,5-diethoxycarbonyl-1,4-dihydrocollidine (DDC) ingestion causes hepatocyte regeneration and a switch to a neo-natal or fetal splicing program (55). This is accompanied by reductions in splicing factor ESRP2, hnRNPH, hnRNPC, and CELF1 protein expression and increases in the MBNL1, PTBP1, hnRNPA1, and SRSF1 protein expression. As a consequence, livers showed alterations in RNA splicing, with exon skipping/retention being the predominant mode. Gene ontology analysis indicated that mRNA processing and spliceosome regulation of splicing were enriched with these alternatively spliced genes. Downregulation of ESRP2 was sufficient to phenocopy DDC treatment (see below).

Not all studies have found changes in the splicing machinery. A number of earlier studies did not report alterations in splicing factor expression. A study carried by Teufel et al. (28) compared gene expression in liver samples from patients at different stages of NAFLD with mouse models of NAFLD. Liver tissues of 25 NASH patients, 27 NAFLD patients, 15 healthy obese patients, and 39 controls were collected, in addition to liver tissues from nine NAFLD mouse models [mice on a high-fat diet (with or without fructose), mice on a Western-type diet, mice on a methionine- and choline-deficient diet, mice on a high-fat diet given streptozotocin, and mice with disruption of *Pten* in hepatocytes]. They found that, although there was very little overlap of gene expression profiles in NAFLD liver tissues between human and mouse, at the pathway level the gene expression patterns in livers of mice with NAFLD due to high-fat diet feeding closely matched the human liver profiles, especially pathways associated with lipid metabolism. Analysis of the human samples uncovered 65 and 177 differentially expressed genes in the NAFLD and NASH groups, but only 12 genes in the healthy obese group. Although splicing factor expression was not significantly altered in the human samples, *Hnrnpab*, *Hnrnpl, Rbm4, Rbm4b, Rbm42, Srsf2*, and *Srsf5* were altered in the mouse MCD and HFD NASH models.

Arendt et al. (27) profiled liver expression in 20 patients with simple steatosis, 19 with NASH and 24 healthy controls by microarray and identified 556 genes altered in the NASH group, while 530 genes were altered in the simple steatosis group and only 22 genes were different between the steatosis and NASH groups. While they were able to show enrichment of genes for fibrosis, inflammation, oxidative stress and lipid metabolism, the dataset did not show enrichment of genes involved in RNA splicing.

Almanza et al. (56) performed transcriptional profiling by RNAseq in SAMP6 mice fed a high-fat diet to induce NAFLD. The authors identified a pre-fibrotic and pre-malignant gene signature in the 350 differentially expressed genes in this mouse model, but splicing factors were not found to be altered.

Kristiansen et al. (57) used a high-cholesterol (2%), high trans-fat (44%) diet to induce NASH in C57BL/six mice compared to obese db/db mice. Although they reported 868 genes induced and 510 genes repressed in their NASH model, the data were not provided so it was not possible to determine whether any splicing factors were among the genes altered.

Similarly, Van Koppen et al. (58) performed RNAseq on livers from LDL-R KO mice fed a high-fat diet to induce NASH. While they reported fibrosis, inflammation, and lipid metabolism signatures, they did not provide the data, so it was again not possible to determine if any splicing factors were altered.

## Contribution of Individual Splicing Factors to Liver Disease

Given the dysregulation of the RNA splicing machinery that has been observed in human and mouse fatty liver disease, a number of studies have looked at the role of individual splicing factors in the liver using mouse genetics (**Table 2**).

**Table 2:** Genetic manipulation of splicing factors implicated in liver disease.

Protein	Class	Model	Phenotype	Ref
**SRSF7**	SR protein family	Homozygous knockout	Embryonic lethal.	(5)
		Heterozygous knockout	Impaired hepatocyte differentiation and maturation.	(5)
**SFRS10**	SR like protein family	Heterozygous knockout	Increased lipogenic gene expression, VLDL, hypertriglyeridemia.	(30)
**SRSF3**	SR protein family	Hepatocyte knockout	Impaired hepatocyte maturation, disrupted glucose and lipid metabolism, HCC.	(65, 66)
		Hepatic SRSF3 stabilization	Reduced liver fibrosis and inflammation.	(67)
**SLU7**	SLU7 family	Hepatocyte knockdown	Impaired glucose and lipid metabolism.	(68)
**NONO**	DBHS protein family	Homozygous knockout	Impaired glucose tolerance, reduced hepatic glycogen, increased fat catabolism, less fat accumulation.	(69)
**ESRP2**	RBM family	Homozygous knockout	Increased inflammatory cytokines, promote adult-to-fetal reprogramming.	(70, 71)
**SRSF1**	SR protein family	Hepatocyte knockout	No liver phenotype. No assessment of splicing.	(72)
**SRSF2**	SR protein family	Hepatocyte knockout	Severe liver injury, mouse early death, cholesterol and bile acid accumulation, hypoglycemia.	(72)
**A1CF**	HNRNP family	Hepatocyte knockout	Improved glucose tolerance. Less hyperglycemia, hepatic steatosis and obesity. Altered splicing.	(73)
**RBM15**	SPEN family	Homozygous knockout	Suppressed hepatic maturation, liver failure.	(74)
**PRPF6**	Leucine zipper family	HCC cell line knockdown	Inhibited cell proliferation in vitro and xenograft HCC tumor growth.	(75)
**MTR4**	SR protein family	HCC cell line knockdown	Reduced cell proliferation, suppressed tumor growth, decreased glycolytic, increased oxidative phosphorylation. Altered splicing.	(76)
**PTBP3**	PTB family	HCC cell line knockdown	Inhibited cell proliferation and metastasis.	(77)
		HCC cell line overexpression	Promoted cell proliferation and metastasis.Altered splicing.	(77)
**MBNL3**	Muscleblind family	HCC cell line knockdown	Inhibited cell proliferation, induced apoptosis, inhibited tumorigenesis. Altered splicing.	(78)
		HCC cell line overexpression	Promoted cell and tumor growth.	(78)

### SFRS10

SFRS10 (also known as TRA2b) belongs to SR-like protein family (79) and was identified as being reduced in the liver and muscle of obese individuals in the paper by Pihlajamaki et al. (30). Like many other SR proteins, the level of SFRS10 protein expression is regulated by a negative feedback loop. SFRS10 binds to its own exon 2 and promotes exon inclusion, generating an mRNA isoform that is subject to non-sense-mediated decay and unable to be translated into protein. When SFRS10 levels decline, exon 2 is skipped and the mRNA isoform encoding full length SFRS10 is produced (80). SiRNA-mediated SFRS10 knockdown increased the expression of several lipogenic genes and increased lipogenesis *in vitro*. Despite the negative feedback regulation, SFRS10 heterozygous mice showed increased expression of lipogenic gene expression in the liver, increased VLDL secretion and hypertriglyceridemia *in vivo* (30).

### SRSF3

SRSF3 (also known as SRp20) is the smallest member of the SR protein family and was previously identified as a regulator of inclusion of exon 11 in the insulin receptor mRNA, which modulates the affinity of the receptor for IGF2. Sen et al. (65) showed that genetic loss of SRSF3 in hepatocytes impaired hepatocyte maturation and disrupted glucose and lipid metabolism. Furthermore, the SRSF3 knockout mice all developed HCC with aging (66). SRSF3 was found to be downregulated in human HCC samples, and recent studies showed SRSF3 was also reduced in NAFLD, NASH and cirrhosis liver samples in both human and mouse, and as a consequence, changes in the splicing of known SRSF3 target genes were observed (*FN1, MYO1B, INSR, SLK*) (10, 81). The increase of Slk spliced isoform and decrease of Dgkd and Insr spliced isoforms have also been detected in mouse livers with inflammation and fibrosis (10). Although SRSF3 protein levels were decreased in fatty liver disease, the levels of its mRNA and the ratio of SRSF3 mRNA isoforms did not change. The level of SRSF3 protein in the liver was determined by proteosomal degradation in the cytoplasm and was controlled by covalent attachment of NEDD8 on lysine 11 (67). Preventing SRSF3 degradation by inhibiting neddylation prevented NAFLD progression in mice, consistent with the fact that the inhibition of neddylation pathway was shown to reverse liver fibrosis *in vivo* (67). Thus, destabilization of a splicing factor under lipid overload is able to trigger liver disease progression. SRSF3 is also implicated in hepatitis B virus pathology as the HBx protein sequesters SRSF3 in the cytoplasm in a 14-3-3*β* complex, which has been shown to enhance Ras/FOXO4 signaling through increased expression of CCDC50S (82).

### SLU7

SLU7 is a splicing factor that ensures the correct selection of the 3′ splice site (83), and its expression was downregulated in patients with cirrhosis (84). Elizalde et al. (68) analyzed the splice events that occurred in HCC cells with downregulated SLU7 and found the most influenced category of genes was RNA post-translational modification. Knockdown of SLU7 in human liver cells and mouse liver impaired glucose and lipid metabolism, and the knockdown mice were unresponsive to normal feeding and fasting. SLU7 was more recently shown to be essential for maintaining genome integrity by suppressing the inclusion of exon 4 in the *SRSF3* mRNA. This transcript is normally subject to non-sense-mediated decay but if translated gives rise to a truncated form of SRSF3 and causes intron retention in sororin mRNA and defects in sister chromatid cohesion and hence, DNA damage (85). In contrast to cirrhosis, SLU7 was shown to be elevated in alcoholic steatohepatitis in humans, and SLU7 knockdown prevented oxidative stress and liver damage in alcohol-treated mice. So, these studies suggest that both overexpression and loss of SLU7 are detrimental to liver function.

### NONO

The RNA binding protein NONO (non-POU domain-containing octamer binding) belongs to the Drosophila Behavior Human Splicing family and binds primarily to introns within pre-mRNAs (86). NONO is an RNA binding protein that forms a heterodimer with SFPQ, a splicing factor that has been shown to be decreased in obesity. Using RNA-immunoprecipitation and sequencing (RIP-seq) NONO bind sites were found to be enriched in metabolic and circadian genes especially after feeding. NONO regulated glucose-responsive genes, including *Gck* and *Glut2*, in the liver post-transcriptionally. NONO-deficient mice had impaired glucose tolerance and reduced hepatic glycogen, were lean and stored less fat, and exhibited increased fat catabolism during the light phase (69). Viral overexpression of NONO improved the glucose tolerance in the NONO-deficient mice. Supporting a role in liver disease, NONO was also found to be elevated in livers from mice on high-fat diet (10). NONO-SFPQ is also the target for the lncRNA Morrbid and modulates *Nras* splicing (87). The NONO and SFPQ genes are frequently co-expressed in HCC where they promote the inclusion of exon12a in the bridging integrator 1 (BIN1) gene causing the expression of the BIN1-L isoform that binds and stabilizes PLK1 (88).

### ESRP2

Epithelial splicing regulatory protein 2 (ESRP2) belongs to the RBM family of RNA-binding proteins and was originally identified as an epithelium-specific splicing regulator (89). A more recent study showed ESRP2 controlled the neonatal-to-adult shift of alternative splicing in the liver (70). Furthermore, Hyun et al. (71) found ESRP2 was suppressed in severe alcoholic hepatitis (ASH) in both humans and mouse models. They further showed that the release of inflammatory cytokines (TNF-α, IL-1β) by excessive alcohol ingestion reprogramed adult hepatocytes into fetal-like cells by suppressing ESRP2. Indeed, depleting ESRP2 exacerbated alcohol-induced steatohepatitis in mouse models. As inflammatory cytokines are involved in liver injury in many disease settings (90–92), ESRP2 suppression and adult-to-fetal reprogramming were also observed in a carbon tetrachloride (CCl_4_)-induced liver fibrosis model (71). Downregulation of ESRP2 activates a neonatal splicing program and causes exon skipping in the *Yap1* and *Tead1* genes, rewiring Hippo signaling and supporting progenitor cell proliferation upon liver injury (55). Altered ESRP2 expression has also been found in human HCC samples (93), so it is reasonable to expect that ESRP2-mediated splicing plays a role in liver disease as inflammation is a marker of progression from NAFLD to NASH (94).

### SRSF2

SRSF2 (also known as SC35) also belongs to the SR protein family and binds exonic splicing enhancers (95, 96). Cheng et al. (72) reported that hepatocyte-specific deletion of SRSF2 caused severe liver injury and early death in mice. RNA-Seq analysis identified SRSF2-regulated cell death and stress-related alternative splicing events, including *Becn1*, *Mfge8, Trp53inp*, and *Trp53inp2.* Furthermore, inactivation of SRSF2 caused hepatic metabolic disorders by controlling expression of transcription factors responsible for energy homeostasis and bile acid metabolism, including *PPARα, C/EBPα, SREBF1c, and NR1I3*, that led to cholesterol and bile acid accumulation in the SRSF2-KO mice. Loss of SRSF2 also decreased expression of metabolic genes such *Ebp, Baat*, *Slc27a5*, resulting in hypoglycemia indicating an essential role of SRSF2 in hepatic metabolism. Interestingly, the few *Srsf2* knockout mice that did not die from liver failure showed impaired hepatocyte maturation, activation of hepatocyte progenitor cells, and eventually developed HCC (97). Deletion of a related SR protein SRSF1 (SF2/ASF) did not result in this phenotype, so the effects are specific and not related to global effects on RNA splicing. Overexpression of SRSF2 has been observed in HCC and knockdown of SRSF2 in human hepatoma cells prevents tumor growth (98).

### SRSF7

SRSF7 (also known as 9G8) is closely related to SRSF3 (99). A study performed by Peng et al. (100) profiled mouse liver transcriptomes during liver development, and SRSF7 expression was shown to decrease during liver maturation. This finding was further supported by Jam et al. who analyzed hepatocytes from juvenile and adult mice (101) and found SRSF7 was expressed more highly in juvenile hepatocytes. Depletion of SRSF7 led to premature maturation, whereas forced expression of SRSF7 suppressed cellular senescence *in vitro*. SRSF7 depletion also impaired cellular anabolism and increased glycolysis consistent with a more fetal-like state. SRSF7 knockout mice also exhibited suppression of juvenility-associated genes in hepatocytes, including *Igf2*, which functions as an enhancer of body growth (5). Thus, SRSF7 is essential for hepatocyte differentiation and maturation.

### A1CF

A1CF (also known as APOBEC1 complementation factor) belongs to the HNRNP family of RNA binding proteins. It was originally identified as an essential component of the ApoB editing complex but recent reports have shown that it is dispensable for RNA editing (102–104). Mice lacking A1cf expression in the liver exhibit improved glucose tolerance and are protected from fructose-induced hyperglycemia, hepatic steatosis, and obesity. The mice have altered RNA splicing of 84 genes including Gk and Khk, and PAR-CLIP studies indicated that A1CF binds to a UGGG sequence and competes with HNRNPH to regulate splicing of various RNA transcripts (73).

### RBM15

RNA binding motif protein 15 (RBM15) belongs to the SPEN family and determines cell fate of many tissues (105). RBM15 binds to RNA to regulate post-transcriptional modifications (106) such as alternative RNA splicing, polyadenylation, and protein translation. RBM15 is not only essential for megakaryocyte differentiation (107), but also indispensable for liver development (74). Hu et al. (74) found RBM15 was expressed in the liver during its differentiation, and depletion of RBM15 specifically suppressed hepatic maturation and caused liver failure but did not affect hepatocyte proliferation and apoptosis. More studies are needed to understand the role of RBM15 in hepatic maturation and liver diseases.

### PRPF6

Pre-mRNA processing factor 6 is a splicing factor involved in spliceosome formation. Depletion of Rpr6 inhibits cell proliferation and HCC tumor growth potentially by upregulating the expression of the androgen receptor splice variant 7 (AR-V7) (75).

### MTR4

MTR4 is an RNA helicase that is present in the TRMAP complex that targets incorrectly processed transcripts for degradation by the nuclear exosome (108, 109). Knockdown of MTR4 expression in HCC cells causes changes in alternative splicing predominantly through exon skipping (76). The authors demonstrated that the glycolytic enzymes Glut1 and Pkm2 are two MTR4 targets, and knockdown of MTR4 increases splicing of the Glut1b and Pkm1 isoforms, causing a metabolic switch from glycolysis to oxidative phosphorylation. Mechanistically, MTR4 acts by recruiting the poly-pyrimidine tract binding protein PTBP1 to 3′ splice sites.

### PTBP3

The polypyrimidine tract binding protein 3 (PTBP3) is overexpressed in HCC and regulates alternative splicing at the 3′ end of the lncRNA NEAT1. This lncRNA controls p53 and CCND1 signaling and hence proliferation (77).

### MBNL3

The muscle blind protein 3 (MBNL3) is expressed highly in fetal liver and is re-expressed in HCC. Transcriptomic analysis of SMMC-7721 HCC cells with *Mbnl3* knockdown revealed 527 MBNL3-dependent alternative splicing events (78). The authors showed that MBNL3 induces exon 4 inclusion in the lncRNA PXN-AS1 that is transcribed from the anti-sense strand of the paxillin (PXN) gene. These anti-sense transcripts have different effects on PXN mRNA translation, with the short isoform inhibiting translation but the long isoform preventing mRNA degradation by miR-24. Paxillin is a focal adhesion protein that promotes tumor cell proliferation, and the authors demonstrated that the oncogenic effects of MBNL3 overexpression are mediated by changes in PXN translation.

## Contribution of Individual Splice Variants to NAFLD

Alternative splicing of mRNA can alter the sequence of the encoded protein. This can alter the biochemical properties of the protein, the intracellular localization, the stability, the ability to be regulated by post-translational modifications, or interactions with other proteins (110). In extreme cases, such as the *Bcl-X* gene, alternative splicing can generate isoforms with antagonistic activity (111). Changes in splicing factor expression can alter the splicing of hundreds of target genes that could potentially be responsible for the observed phenotype. So, in this section, we will look at individual splice variants in target genes and their possible role in fatty liver disease.

### KLF6

Kruppel-like factor 6 (KLF6), a member of the Kruppel-like family, was identified as an activator of several genes involved in the development of liver fibrosis (112), and its expression is increased during progression to fibrosis in a rat NASH model (113). Genetic association studies have also shown that a SNP located in the first intron is associated with NAFLD. The functional SNP creates a novel binding site for a splicing factor SRp40 that alters the splicing of the KLF6 pre-mRNA allowing production of a shorter isoform. While the KLF6 full length isoform was increased in NAFLD patients with more advanced disease, the alternative spliced isoform of KLF6 enhanced by the SNP was associated with reduced fibrosis in NAFLD. Miele et al. (114) found that the shorter KLF6 isoform was anti-fibrogenic and could abrogate the induction of *α*-smooth muscle actin and type 1 collagen mRNAs *in vitro* by full length KLF6. Moreover, another study (115) revealed that the alternatively spliced variant of KLF6 lowered the hepatic insulin resistance and blood glucose by reducing glucokinase expression.

### PPAR*γ*

The peroxisome proliferator-activated receptor family (PPARs) consists of three genes: PPAR*α*, PPAR*β/δ* and PPAR*γ*, with a highly conserved structure (116). The PPARs regulate genes involved in multiple processes, such as fatty acid uptake and oxidation, lipid metabolism and inflammation (117). PPAR*α* and PPAR*β/δ* play a role in lipid catabolism, while PPAR*γ* regulates lipid anabolism and is essential for induction of adipogenesis (118). PPAR*γ* has multiple alternatively spliced transcripts but two major protein isoforms: PPAR*γ*1 and PPAR*γ*2. Many transcripts encode PPAR*γ*1, but only the PPARG-201 transcript, which initiates from a downstream promoter, encodes the PPAR*γ*2 protein (119). Though PPAR*γ*1 expression is very low in normal liver, PPAR*γ*2 expression is significantly elevated in terms of both mRNA and protein levels in the livers of obese mice compared to the wide type mice. Furthermore, the increased PPAR*γ*2 expression was positively correlated with liver steatosis in obese patients and with insulin resistance in mice (120–122). Given PPAR*γ*2’s role in induction of adipogenic genes, the elevated expression in the liver is consistent with the induction of steatosis in obesity. PPAR*γ*2 activation induced lipogenic genes (including *ADRP, SREBP-1*, and *FAS*) and promoted *de novo* lipogenesis resulting in lipid accumulation in hepatocytes (123–125).

### INSR

The insulin receptor (INSR) is a tyrosine kinase receptor that mediates both the metabolic and mitogenic effects of insulin (126). The association between INSR and NAFLD has been widely investigated since Marchesini et al. first demonstrated that NAFLD patients had reduced insulin sensitivity and impaired hepatic glucose production (127). INSR has two isoforms due to alternative splicing of exon 11: INSR-A and INSR-B (128, 129). INSR-A lacks exon 11 and binds both insulin and IGF-2 with high affinity, but INSR-B that contains the additional 12 amino acids encoded by exon 11 only binds insulin with high affinity (130). The expression of these two different isoforms is regulated at both mRNA transcription and post-transcription levels (128, 129). In many cancers, INSR is overexpressed and the A:B ratio increased (129). Kumar et al. (81) have reported that the splicing of the INSR is altered in patients with NAFLD, NASH, and cirrhosis and in mice on high-fat diet or NASH diets, with increased expression of INSR-A. In pre-clinical studies, Lopez-Pastor et al. reported that AAV expression of INSR-A or INSR-B significantly reduced the NAFLD activity score (NAS) and improved insulin secretion, but did not affect body weight or glucose tolerance in mice on a high fat diet (131). Interestingly, INSR-A improved insulin sensitivity and increased glucose uptake into liver and muscle. Similar studies in a liver *Insr* knockout mouse showed that INSR-A was more effective at ameliorating glucose intolerance (132). The two receptors are known to signal differentially and INSR-A and INSR-B showed different effects on gene expression as has been reported in pancreatic beta cells (133). Thus, altered INSR splicing in liver could potentially alter NAFLD progression.

### LPIN1

LPIN1 is a member of the lipin gene family that dephosphorylates phosphatidic acid to diacylglycerol in the penultimate step in triglyceride metabolism (134). LPIN1 gene encodes two mRNA isoforms, lipin-1*α*, lipin-1*β* by alternative mRNA splicing (135). Lipin-1*β* includes exon 6 compared with lipin-1*α* (35). The two isoforms of lipin-1 differ in expression pattern, subcellular localization, and function (35). During the adipocyte differentiation, lipin-1*α* decreases, and lipin-1*β* increases. In mature adipocytes, lipin-1*α* localizes in nucleus but lipin-1*β* is primarily cytoplasmic. Unlike lipin-1*α*, lipin-1*β* expression leads to induction of lipogenic genes (35). The expression of lipin-1*β* mRNA but not lipin-1*α* increased in the livers of NASH mice induced by choline-deficient diet (136). Pihlajamaki et al. (30) reported that SFRS10 regulates lipin-1 mRNA splicing by binding to exon 6 causing its inclusion. SFRS10 levels are reduced in the livers of obese mice and obese humans and, although overall expression of lipin-1 did not change, the lipin-1*β*/*α* ratio increased. Yin et al. (137) demonstrated that lipin-1*β*/*α* is also increased in alcoholic fatty liver disease (AFLD) in mice. They also showed that alcohol reduces SIRT1 expression in mouse liver which in turn reduces SRSF10 expression. Genetic deletion of SIRT1 in hepatocytes causes hepatic steatosis suggesting this may be causative in AFLD (138). The downregulation of SIRT1 was also observed in obese human subjects, and LPIN1 splicing was altered, but paradoxically, SRSF10 levels were not changed (139). More evidence is needed, therefore, to fully establish the SIRT1–SFRS10–LIPIN-1 axis in fatty liver in humans.

### TF

Tissue factor (TF) is produced by the liver and is required for blood coagulation (140). An alternatively spliced form of TF is found in the plasma of patients with chronic liver disease including liver fibrosis, cirrhosis and HCC. This isoform lacks exon 5 leading to premature termination of translation in exon 6, the last exon (141). The resulting protein lacks the transmembrane domain that anchors TF in the plasma membrane as TF acts as an angiogenic factor by stimulating integrin signaling, triggering proliferation and tumor cell metastasis.

### PDSS2

The prenyldiphosphate synthase subunit 2 (PDSS2) is a key factor in coenzyme Q10 synthesis. Six splicing variants are produced, but only full length PDSS2 is catalytically active, the other five variants showing loss of function. Loss of PDSS2 function causes a shift from mitochondrial respiration to aerobic glycolysis and increased proliferation of HCC cells that can only be restored by the full length Pdss2 isoform (142). Knockdown of PDSS2 in MIHA immortalized liver cells caused chromosomal instability and transformation.

## Conclusion and Future Perspectives

It is generally accepted that alternative RNA splicing plays an important role in fine tuning gene expression and cellular function in various tissues and contexts. There is also evidence that changes in RNA splicing may be involved in the pathogenesis of obesity in various tissues (143). In the liver, changes in RNA splicing have been documented during development and maturation of hepatocytes, but studies showing a causal relationship are rare. In 2017 (17), we reviewed the status of the field and the published studies of alternative splicing in the liver. Since then, a number of large human studies have been published so we have updated our review to include these and other new studies that further support the concept that alternative splicing is an early feature of liver disease. While RNA splicing changes have been documented in HCC (8–16) and reviewed elsewhere (144–146), most studies in early liver disease, NAFLD or NASH, have focused on total mRNA changes rather than changes in individual mRNA isoforms and alternative splicing (48–50). These studies, however, can be informative as changes in the expression of splicing machinery components can be indicative of changes in RNA splicing. The idea that these changes may be causative for, rather than the result of, liver disease is supported by accumulating evidence from genetic manipulation of individual splicing factors or isoform variants in mice that contribute to liver disease. It is worth noting that most of the altered mRNAs or splicing factors are associated with lipid metabolism which is not unexpected given the common steatotic phenotype. Beyond that, gluconeogenesis and fibrosis are also disease processes that may be influenced by alternative splicing, which may contribute to disease progression, inflammation and fibrosis. Given the potential role of individual mRNA isoforms in liver disease, further investigation into the extent of splicing dysregulation in liver disease, the splicing factor target networks involved, and the function of the alternatively spliced isoforms is clearly required. Additionally, as the transcriptome profiles between humans and mice with NAFLD have been shown to differ, it would be important to compare alternative splicing patterns in human liver and mouse models of liver disease. This is particularly important as studying hepatic-specific KO mice and splice variants is a powerful tool for functional evaluation of alternative splicing variants *in vivo*. Functional assessment is essential as it is not possible *a priori* to predict the effect of a splice variant on protein activity. Unlike transcriptome profiling where an increase in gene expression generally leads to an increase in protein expression and activity, this is not the case for alternative splicing. Indeed, there are many examples of splice variants having antagonistic activities. Gene-to-gene mapping for transcriptome studies is possible but mapping alternative splicing events is more problematic. There is currently no widely accepted method to name splicing events, and the output from most software programs is not compatible with cross-comparisons. The development of standardized formats and nomenclature in the splicing field will be required to enable the use of animal models to predict human disease.

The recent studies provide a strong rationale for the development and testing of novel therapeutic strategies targeting specific isoforms based on an understanding of alternative splice variants in NAFLD. Small molecule splicing inhibitors have been developed to interfere with the activity of the spliceosome and are being tested in cancer models (147). Yet these general inhibitors are unlikely to be useful in other diseases due to broad effects to inhibit all splicing. A more promising approach is the development of splice-switching anti-sense oligonucleotides (ASO) for diseases related to RNA mis-splicing, thus minimizing toxic side effects (148, 149). Although anti-sense oligonucleotides (ASO) delivery is a challenge, recent advances in the modifications have improved the stability, affinity (150, 151) and specificity (152). For example, the *BCL-X* gene has two major isoforms: antiapoptotic *BCL-XL* and proapoptotic *BCL-XS*. ASO can induce a shift from *BCL-XL* to *Bcl-XS* in human hepatic stellate cells, which are the major producers of fibrotic ECM, showing that *BCL-X* ASO is a potential therapy for liver fibrosis (153). Other ASOs have been proved to protect mice from NAFLD through aiming at gene silencing (154, 155). Alternative therapeutic approaches include the development of modified U1 snRNAs that target specific splicing mutations (156). These have been used successfully in preclinical studies in familial dysautonomia (157), hemophilia B (158), and spinal muscular atrophy (159). Development of such targeted therapeutics for liver disease will require further studies on alternative splicing in NAFLD and the role of individual splice variants.

## Author Contributions

PW wrote the first draft. MZ helped revise the manuscript. NW edited and finalized the manuscript. All authors contributed to the article and approved the submitted version.

## Funding

This work was funded partly by a Veterans Affairs Merit Review awards (I01BX000130 and I01BX004848) and Senior Research Career Scientist Award (IK6BX005224), and partly by NIH grants (CA196853, AI25860, and HL141999) to NW.

## Conflict of Interest

The authors declare that the research was conducted in the absence of any commercial or financial relationships that could be construed as a potential conflict of interest.
